# Disparities in PrEP use and unmet need across PEPFAR-supported programs: doubling down on prevention to put people first and end AIDS as a public health threat by 2030

**DOI:** 10.3389/frph.2024.1488970

**Published:** 2024-12-13

**Authors:** Trena I. Mukherjee, Mitchell Yep, Megan Koluch, Sisay Alemayehu Abayneh, Gizachew Eyassu, Elizabeth Manfredini, Sara Herbst

**Affiliations:** ^1^ Office of HIV/AIDS, Prevention, Care and Treatment Division, USAID, Washington, DC, United States; ^2^ Bureau of Global Health Security and Diplomacy, President’s Emergency Plan for AIDS Relief (PEPFAR), Office of Program Impact, Monitoring, and Epidemiology (PRIME), U.S. Department of State, Washington, DC, United States; ^3^ PEPFAR Coordination Office, U.S. Embassy Rwanda, Kigali, Rwanda; ^4^ USAID/Ethiopia, Health Office, Infectious Disease Team, U.S. Embassy Ethiopia, Addis Ababa, Ethiopia

**Keywords:** PrEP-to-need ratio, PEPFAR, PrEP, HIV prevention, key populations, AGYW, HIV

## Abstract

**Background:**

In 2023, an estimated 1.3 million people newly acquired HIV. In the same year, 3.5 million individuals received pre-exposure prophylaxis (PrEP), falling short of the UNAIDS target of 21.2 million by 2025. With over 90% of global PrEP programming supported by PEPFAR, a better understanding of disparities in PrEP provision is needed to inform PEPFAR's approach to reach and deliver prevention services and achieve UNAIDS 95-95-95 goals in all populations by 2025. The objective of this paper is to assess unmet PrEP need in PEPFAR-supported countries.

**Methods:**

We analyzed FY2023 Monitoring, Evaluation, and Reporting (MER) results from 48 PEPFAR-supported countries to calculate PrEP-to-need ratios (PnR) by geography and population. PnR offers an ecological measure to identify disparities and missed opportunities for PrEP programming. PnR was calculated as the ratio of PrEP users to the number of positive HIV tests. PrEP users are defined through new initiations (PrEP_NEW) and re-initiations or continuation (PrEP_CT). HTS_TST_POS measures the number of positive HIV tests and was used as a proxy for new diagnoses. PnR was also calculated using Naomi-estimated 2023 HIV incidence, where available. A higher PnR indicates more PrEP users relative to PrEP need in a population.

**Results:**

In FY23, 1,760,888 people initiated PrEP, and 1,736,144 people tested positive for HIV. PnR ranged from 0.12 (India) to 6.46 (Brazil), and 19 (40%) countries had fewer PrEP users than positive HIV tests (PnR <1.0). By population, people 15–24 years old, people who inject drugs, and transgender populations had the highest median PnR. When examining estimated HIV incidence, Mozambique and South Africa reported lower than average PnR and higher than average HIV incidence.

**Conclusion:**

PrEP use relative to population need varied greatly by country and subpopulation across PEPFAR programs, suggesting a need for greater advocacy, inclusivity, accessibility, and integrated prevention programming. PnR may be a useful indicator of population PrEP coverage and unmet need, and can inform effective, data-driven, and person-centered PEPFAR prevention programming and policies. Tailoring PrEP scale-up strategies by age, sex, key population, and geography is crucial to achieving UNAIDS targets and ending the AIDS epidemic as a public health threat for all by 2030.

## Introduction

While there has been substantial progress towards ending the HIV/AIDS epidemic, we are not on track to reaching the UNAIDS target of less than 370,000 annual new infections by 2025 (1). In 2023, an estimated 1.3 million people newly acquired HIV, with adolescent girls and young women (AGYW), and key populations and their sexual partners accounting for 46% and 55% of all new infections, respectively (2, 3). Furthermore, HIV incidence has declined by 39% since 2010, but there are substantial disparities by population and geography (3). For example, HIV incidence has declined globally by 10% among key populations overall and has increased by 11% and 3% among MSM and TG, respectively (4). In sub-Saharan Africa (SSA), incidence has declined across all populations since 2010; however, outside of SSA, nearly all key populations and their partners experienced substantial increases in incidence (4).

To reach UNAIDS targets of fewer than 370,000 annual infections by 2025, UNAIDS has a goal of ensuring 95% of people at risk for HIV acquisition use appropriate, prioritized, effective combination prevention (3). Pre-exposure prophylaxis (PrEP) reduces the risk of acquiring HIV through sexual transmission by 99% and parenteral (injection drug use) transmission by at least 74% (5–7), and is an effective and essential, evidence-based biomedical component of combination prevention. On a population level, greater PrEP coverage has been associated with decreased HIV incidence, as evident in the U.S., where the highest quintiles of PrEP coverage are associated with the greatest decreases in HIV diagnoses (8); and in Australia, where greater PrEP coverage (>60% of days with PrEP use) is associated with a 79% decrease in HIV incidence (9).

Over 3.5 million people used PrEP in 2023, falling short of the 2025 global PrEP target of 21.2 million people (3). All regions have yet to reach PrEP targets, with Eastern and Southern Africa having made the most progress to date, and Asia and the Pacific region facing the largest gap (3). Substantial scale-up is needed to accelerate progress towards UNAIDS goal to end AIDS as a public health threat by 2030. Yet, PrEP access and PrEP use varies widely by country and by population due to stigma, criminalization, differentiated and simplified service delivery, age of consent, and other policies restricting PrEP eligibility to certain populations (10–12).

The U.S. President's Emergency Plan for AIDS Relief (PEPFAR) added PrEP as a core programmatic requirement in 2021 (13) and contributed to nearly 90% of global PrEP initiations across 49 PEPFAR-supported countries in 2023 (14). A better understanding of PrEP coverage and unmet need is needed to inform PEPFAR's approach to reach and deliver prevention services to support UNAIDS 95-95-95 targets for all populations by 2025. PrEP-to-need ratio (PnR) offers an approach to measuring unmet need. PnR is defined as the ratio of the number of PrEP users to the number of new HIV diagnoses, in a given geographic area or population. PnR has been previously used to identify disparities in PrEP coverage and socioeconomic factors associated with disparities in PrEP use in the U.S. (15, 16). and equity of PrEP access in England (17). PnR has also been used within PEPFAR programming to assess how the COVID-19 pandemic affected PrEP coverage across 21 PEPFAR-supported countries (18), and unmet need among AGYW in Determined, Resilient, Empowered, AIDS-Free, Mentored and Safe (DREAMS) countries (11). Overall, PnR measures how PrEP use compares to the need for PrEP in a given population and will highlight missed opportunities for PrEP coverage within PEPFAR programming. PnR may be a useful proxy measure for PrEP coverage in settings where PrEP uptake and HIV incidence cannot be directly measured (16). This paper aims to assess unmet need for PrEP in PEPFAR supported countries, by country and subpopulation, to inform data-driven and person-centered prevention programs and policies.

## Methods

We utilized quarterly Fiscal Year 2023 (FY23, October 1, 2022 - September 30, 2023) PEPFAR Monitoring, Evaluation, and Reporting (MER) results to describe PrEP scale-up in 48 PEPFAR-supported countries with PrEP service provision (19). Nigeria was excluded due to ongoing data quality improvement efforts in FY23. Because MER reporting only includes de-identified data that is reported in aggregate, this analysis is not considered human subjects research and did not need to undergo ethics review.

### Data analysis

An ecological study was conducted to identify unmet PrEP need in PEPFAR-supported countries. Unmet need is described through PrEP-to-need Ratio (PnR), using previously published methodology (8). Annual PnR was calculated as the ratio of PrEP users to the number of positive HIV tests. PrEP users are defined as the number of PrEP naive persons newly initiating PrEP (PrEP_NEW) and the average number of persons continuing or re-initiating PrEP (PrEP_CT) for each quarter in FY23. The number of persons with a positive HIV test (HTS_TST_POS) was used as a proxy for new HIV infections.

$$\mathrm{Annual}\;\mathrm{Pn}R_{\mathrm{MER}}=\frac{\sum_{i=1}^{4}\mathrm{PrEP}\_{\mathrm{NEW}}_{i}+\frac{1}{4}\sum_{i=1}^{4}\mathrm{PrEP}\_{\mathrm{CT}}_{i}}{\sum_{i=1}^{4}\mathrm{HTS}\_\mathrm{TST}\_\mathrm{PO}S_{i}}$$

A higher PnR indicates more PrEP users relative to PrEP need in a population. Thus, a PnR greater than 1.0 means more PrEP users relative to positive HIV tests, and a PnR less than 1.0 means there are fewer PrEP users relative to positive HIV tests in FY23.

MER-derived PnR estimates are reported by country, age (15+, 15–24 years, 25–34 years, 35–49 years, 50+ years), sex (male, female), and key population subgroup [female sex workers (FSW), men who have sex with men (MSM), people in prisons, people who inject drugs (PWID), and transgender persons (TG)], subject to data availability. Age and sex, and key population subgroups are not mutually exclusive (e.g., some females aged 15–24 years old may also be categorized as FSW); however, key population subgroups are mutually exclusive (e.g., women who engage in sex work and inject drugs are categorized as either FSW or PWID). PnR estimates for each country and population were calculated based on the data reported in FY23 and results are subject to data availability. PnR calculations missing PrEP and/or HIV testing data are not reported in the results. In some instances, PrEP and/or HIV testing data reporting is incomplete, and results should be interpreted with caution (see Supplementary Table S1 footnotes).

HIV incidence estimates come from the Naomi model, which synthesizes multiple subnational data sources to furnish estimates of key indicators for HIV program planning, resource allocation, and target setting (20). HIV incidence data from Spectrum/Naomi were collected using the following parameters: Area Level 0, September 2023, and HIV incidence per 1,000. For Malawi, data for December 2023 were used because there were no estimates for September 2023. Country-level disparities in PrEP use were identified based on the mean of MER-derived PnR estimates and mean HIV incidence. Naomi provides published HIV incidence estimates for 27 PEPFAR-supported countries; therefore, country-level comparisons were restricted to those 27 countries.

Additionally, a supplemental PnR analysis was completed using available new HIV infection estimates from Naomi, in which PnR was calculated as the ratio of PEPFAR-reported PrEP users in FY23 to the Naomi-estimated number of new HIV infections in 2023:

$$\mathrm{Annual}\;{\mathrm{PnR}}_{\left(\mathrm{Naomi}\right)}=\frac{\sum_{i=1}^{4}\mathrm{PrEP}\_\mathrm{NE}W_{i}+\frac{1}{4}\sum_{i=1}^{4}\mathrm{PrEP}\_CT_{i}}{\mathrm{Estimated}\;\mathrm{new}\;\mathrm{infections}\;(\mathrm{Naomi})}$$

Naomi-derived PnR estimates are summarized by country and by age and sex in the Supplementary Table S2. This provides an additional method to calculate PnR, which may be more applicable in countries that do not have PEPFAR-supported HIV programs that report MER data, or in countries where PEPFAR support is limited to certain geographies or has limited reach due to national or other donor support.

## Results

**Figure 1:**
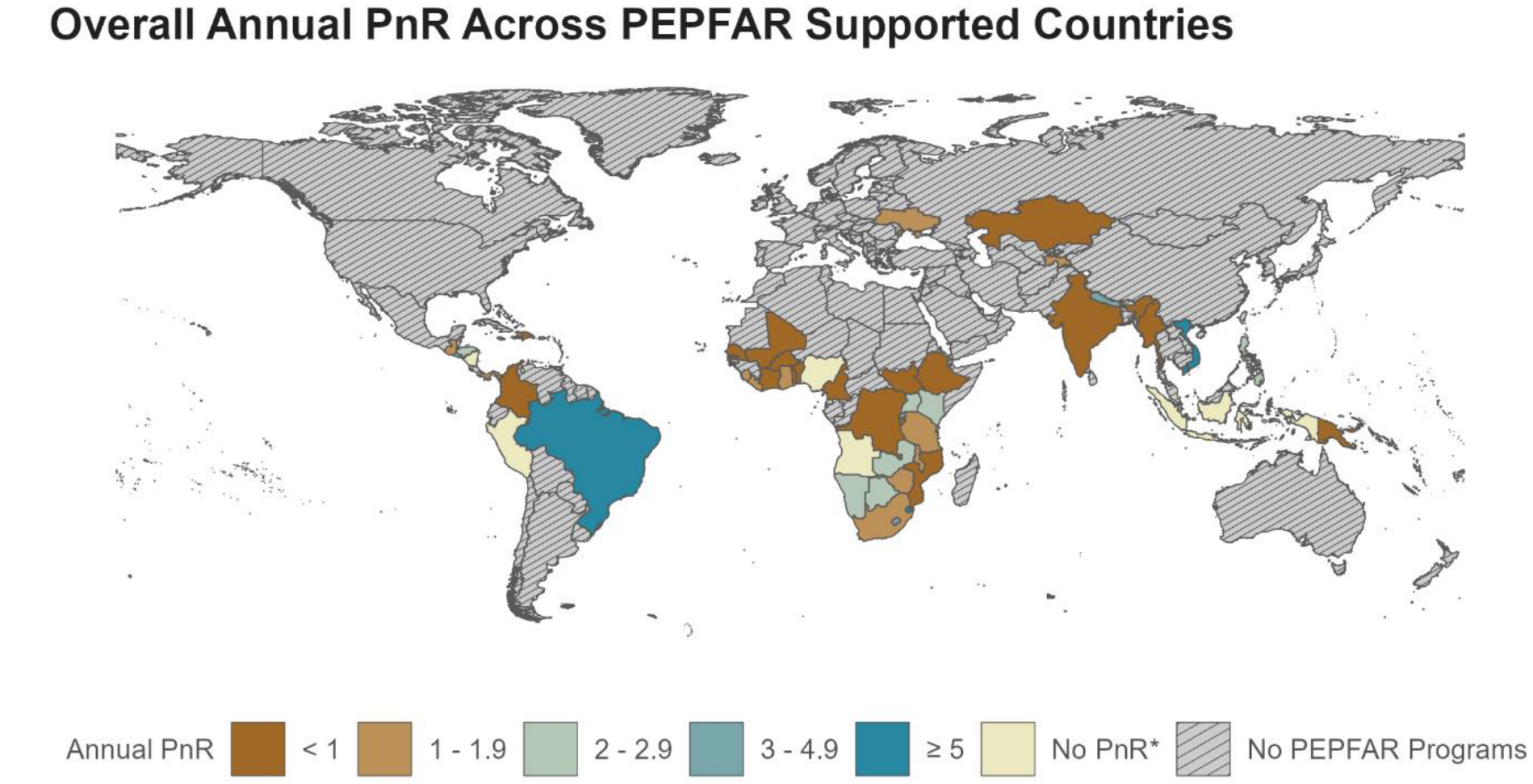
PrEP-to-need ratio (PnR) in PEPFAR-supported countries in FY23. *PnR not calculated due to incomplete PrEP and/or HTS data. Refer to Supplemental Table S1 for all PnR estimates and details on data availability.

In FY23, 1,760,888 people initiated PrEP, an average of 510,551 people continued PrEP, and a total of 1,736,144 people tested HIV-positive across the 48 PEPFAR-supported countries included in the analysis. This equates to an average PnR of 1.31 across all PEPFAR-supported programs, meaning there were 1.31 PrEP users for each positive HIV test. Of the 48 PEPFAR-supported countries with PnR estimates, 19 (40%) countries had a PnR <1.00, indicating fewer PrEP users relative to positive HIV tests in FY23, with varying geographic distribution and need for PrEP scale-up in all regions (Figure 1). Countries with a PnR <1.00, suggesting a need for PrEP scale-up included: Benin, Burkina Faso, Burma, Burundi, Cameroon, Colombia, Cote d'Ivoire, Dominican Republic, Democratic Republic of Congo, Ethiopia, India, Kazakhstan, Laos, Mali, Mozambique, Papua New Guinea, Senegal, South Sudan, and Togo. Across all countries with complete data, the median PnR was 1.19 and ranged from 0.12 in India to 6.46 in Brazil (Supplementary Table S1). By region, Africa accounted for 1,674,282 (95.1%) PrEP initiations with a PnR of 1.29; East Asia & Pacific accounted for 43,402 (2.5%) PrEP initiations with a PnR of 3.82; Eastern Europe and Central Asia accounted for 8,263 (0.5%) PrEP initiations with a PnR of 1.23; Latin America & the Caribbean accounted for 28,512 (1.6%) PrEP initiations with a PnR of 1.17; and South Asia accounted for 6,429 (0.4%) PrEP initiations with a PnR of 0.49.

**Figure 2:**
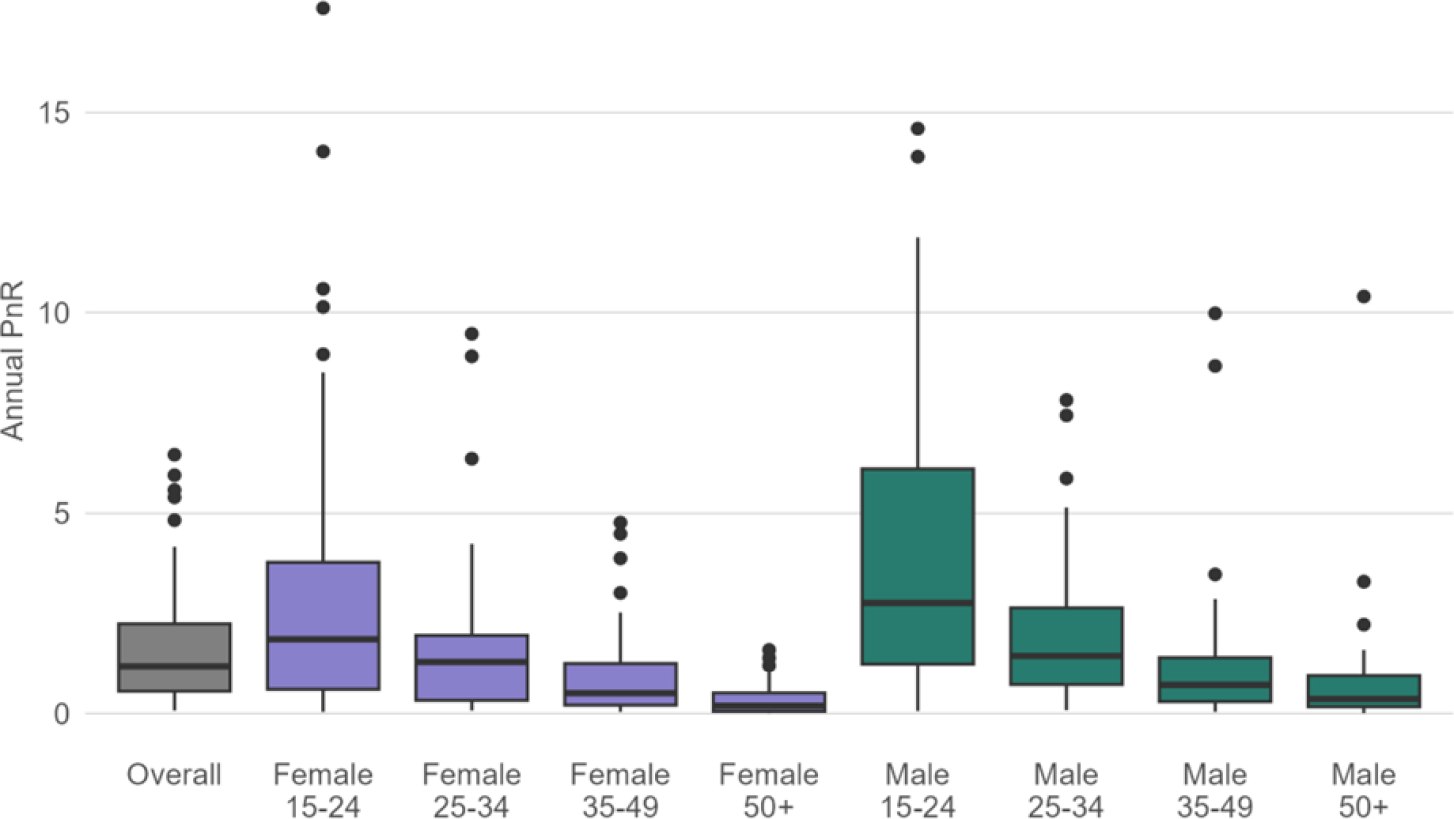
Distribution of MER PrEP-to-need ratio (PnR) in PEPFAR-supported countries by sex and age in FY23.

**Figure 3:**
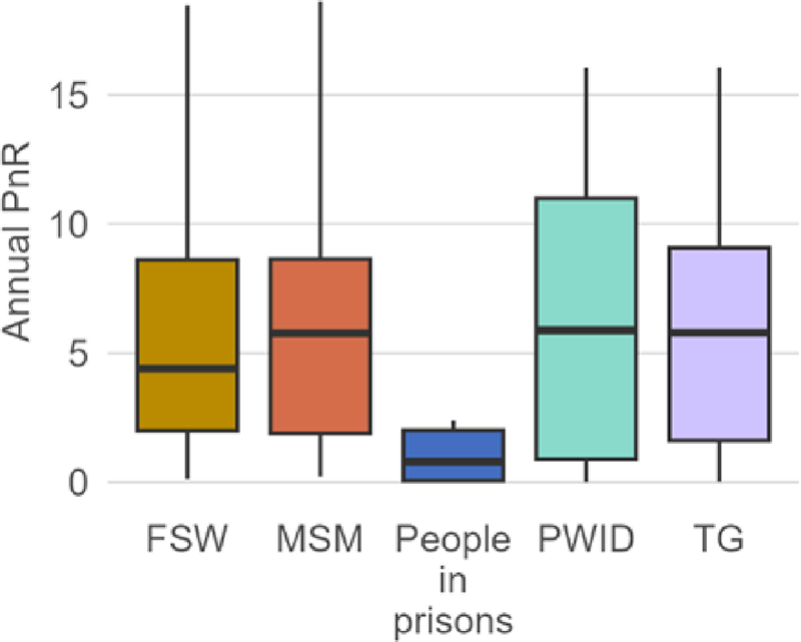
Distribution of MER PrEP-to-need ratio (PnR) by key population in FY23. Outliers beyond the upper and lower limit were removed from this visualization for ease of interpretation across key populations, through the data is included in the box plots. Refer to Supplemental Table S1 for all PnR estimates, including outliers. FSW, female sex workers; MSM, men who have sex with men; PWID, people who inject drugs; TG, transgender.

In FY23, 867,579 adolescents and young people (15–24 years) initiated PrEP. Adolescent girls and young women (aged 15–24 years) had the highest median PnR at 1.87 among all female populations, whereas adolescent boys and young men had the highest PnR at 2.77 among all male populations (Figure 2). Among key populations with available data, 468,407 initiated PrEP in FY23. The median PnR was 4.40, 5.77, 0.80, 5.88, and 5.79, among FSW, MSM, people in prisons, PWID and TG, respectively, with the lowest PnR among people in prisons, and the highest among PWID (Figure 3). Across countries, PnR ranged from 0.13 in Myanmar to 152.88 in Honduras among FSW (*n* = 45 countries); 0.23 in Colombia to 60.63 in Sierra Leone among MSM (*n* = 46); ≤0.02 in Cameroon, Rwanda, and Tanzania to 21.50 in Lesotho among people in prisons (*n* = 13); <0.01 in India to 54.13 in Liberia among PWID (*n* = 26); and 0.02 in Cameroon to 63.72 in Vietnam among TG (*n* = 32).

**Figure 4:**
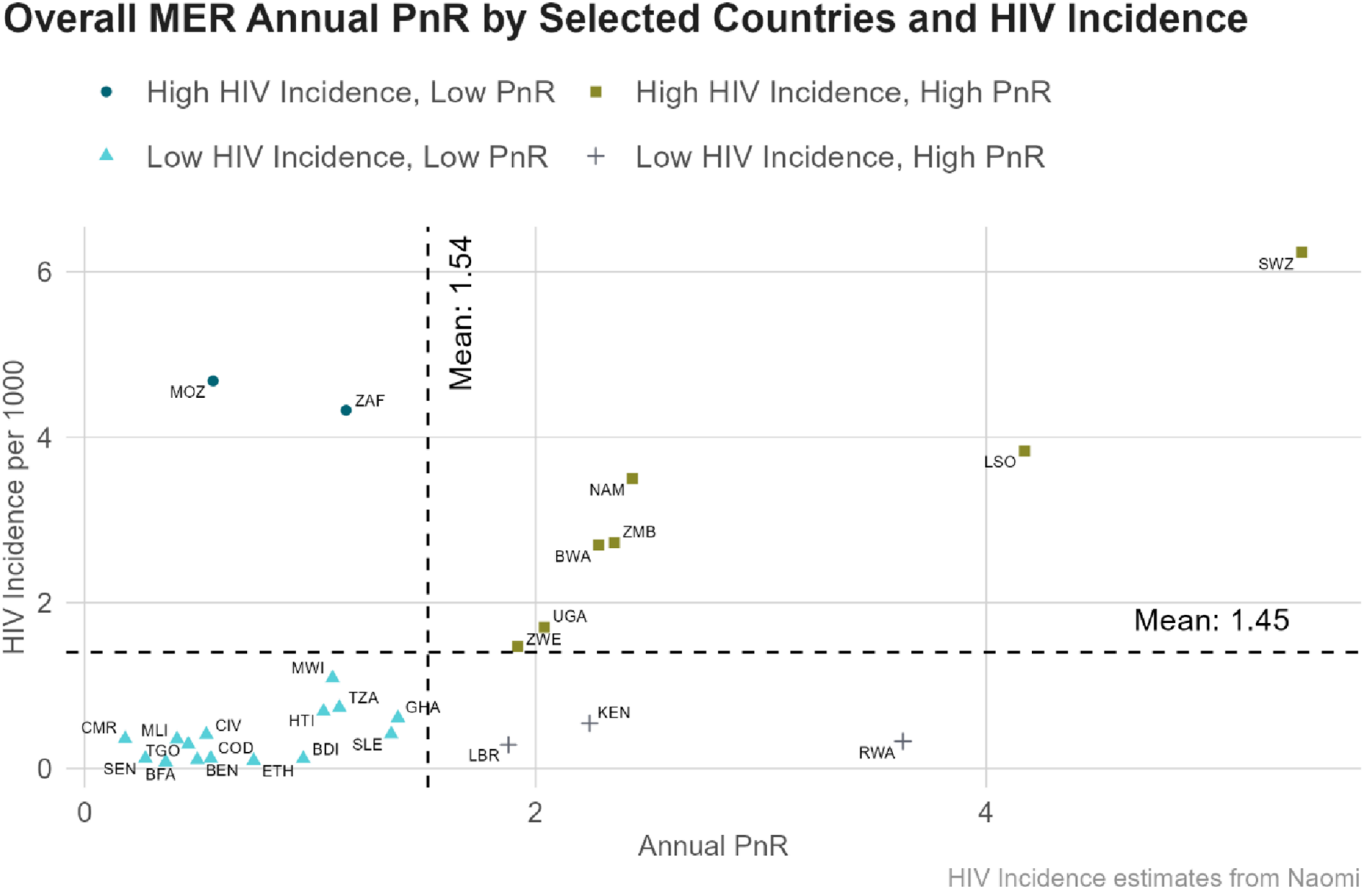
Relationship between HIV incidence and MER PrEP-to-need ratio (PnR) in select PEPFAR-supported countries in FY23. Data is limited to countries with available HIV incidence from the Naomi model. BEN, Benin; BWA, Botswana; BFA, Burkina Faso; BDI, Burundi; CMR, Cameroon; CIV, Cote d'Ivoire; COD, Democratic Republic of Congo; SWZ, Eswatini; ETH, Ethiopia; GHA, Ghana; HTI, Haiti; KEN, Kenya; LSO, Lesotho; LBR, Liberia; MWI, Malawi; MLI, Mali; MOZ, Mozambique; NAM, Namibia; RWA, Rwanda; SEN, Senegal; SLE, Sierra Leone; ZAF, South Africa; TGO, Togo; TZA, United Republic of Tanzania; UGA, Uganda; ZMB, Zambia; ZWE, Zimbabwe.

When examining the relationship between country-level PnR values and estimates of HIV incidence per 1,000 (Figure 4) to categorize countries, Mozambique and South Africa had an estimated HIV incidence higher than the mean (1.45), and PnR lower than the mean (1.54). In contrast, Eswatini, Lesotho, Namibia, Zambia, Botswana, Uganda, and Zimbabwe had estimated HIV incidence and PnR higher than the mean. Liberia, Kenya and Rwanda have HIV incidence lower than the mean, but higher PnR values. All other countries had lower HIV incidence and lower PnR values than the mean.

Finally, PnR estimates derived from MER were compared to PnR estimates derived from Naomi to compare national PnR values using estimated HIV incidence. With the exception of Botswana, Naomi-derived PnR estimates were in the same quartile as MER-derived PnR estimates (Supplementary Figure S1). Botswana moved from high PnR and high HIV incidence using MER-derived estimates, to low PnR and high HIV incidence using Naomi-derived estimates. Additionally, Naomi-derived models had higher PnR values, on average, due to lower HIV incidence, particularly among females (Supplementary Figures S2, S3).

## Discussion

PnR estimates are an ecological construct, which can be used to explore disparities in PrEP provision across populations and geographies. PnR does not assess individual-level factors, however, such as exposure to HIV or access to healthcare, which are also essential for understanding whether PrEP is appropriately targeted to individuals in need. The PnR estimates reported here may be used to guide opportunities to identify areas for further PEPFAR investments into PrEP programming and opportunities for greater advocacy and equitable access to integrated prevention programming. While PnR has been used to evaluate unmet PrEP need in multiple settings (11, 16, 18, 21), there is no globally agreed upon “cut off” for PnR. Instead, PnR estimates can be viewed in relation to each other across or within countries and populations to understand whether PrEP distribution and uptake is meeting need at the population-level. PnR can be one tool to identify geographies or populations for PrEP scale-up, prioritize resources, and measure impact of PrEP on epidemic progress, alongside other measures such as ART coverage and population viral suppression. Overall, a high PnR means that PrEP is widely available and is used by those at-risk for HIV acquisition, whereas a low PnR suggests that PrEP is underutilized by those most at-risk for HIV acquisition.

Our results indicate that on a population-level, PrEP use relative to need varied greatly by region, country, and population across PEPFAR programming in FY23. Forty percent of PEPFAR-supported countries had PnR values <1.0, in which the number of PrEP users were fewer than the number of positive HIV tests. This suggests that significant and urgent PrEP scale-up is needed across nearly all populations in many PEPFAR-supported countries, and targeted scale-up across demographic and key populations in other PEPFAR-supported countries in order to reach the UNAIDS goal of ending the AIDS epidemic as a public health threat for all by 2030.

Although nearly all regions had an average PnR >1.0, East Asia & Pacific reported the highest PnR on average (3.82). Sub-Saharan Africa contributed to the highest proportion of PrEP initiations within PEPFAR (95.1%). According to UNAIDS 2024 data, however, PrEP is not scaling fast enough to reduce new infections, and the current achievement of 3.5 million individuals at-risk for HIV infection using PrEP is far from the 2025 global target of 21.2 million needed for epidemic control (3). Within PEPFAR, overall PrEP use is greater than the number of HIV positive tests, though there is still an urgent need to target PrEP scale-up and expansion using a precision prevention approach in nearly all PEPFAR countries, as indicated by UNAIDS estimates (3). Numerous studies have suggested low PrEP uptake among adolescents, young people and key populations due to ongoing challenges in expanding access and awareness, despite efforts to reach these groups (22). Results reported in this paper, however, show that adolescents and young people (15–24 years) reported highest median PnR, though some countries had PnR values <1.0 among males and females 15–24 years of age. This suggests that PrEP programming in PEPFAR- supported regions are reaching key and priority populations at higher risk for HIV acquisition, consistent with PEPFAR's priorities for youth and young people, and for key populations (23). Furthermore, targeted and tailored prevention interventions for adolescents and young people are still needed to support PrEP uptake and reduce HIV acquisition in some countries. Demographic (age/sex) and key population data are not mutually exclusive in this analysis, and targeted and tailored interventions for young key populations must also be considered.

Globally, key populations are disproportionately affected by HIV, in which the relative risk of acquiring HIV is 9 times higher for sex workers, 23 times higher for MSM, 14 times higher for PWID, and 20 times higher for transgender women than in the general population, due to stigma and discrimination, limited access to healthcare and other structural vulnerabilities (4). Key population MER data were available for FSW and MSM in most PEPFAR countries, but were limited for transgender populations, PWID and people in prisons in several countries. While PnR values were relatively high among most key populations, they were low for people in prisons (median PnR = 0.80) despite PrEP being listed as an essential health intervention by the World Health Organization (24). Findings from this paper suggest relatively high PnR among key populations in PEPFAR-supported countries. However there is still room for substantial growth and PrEP scale-up for key populations at high risk for HIV infection, given that over half of all new infections in 2023 were among key populations and their sexual partners (3).

The relationship between country-level PnR values and estimates of HIV incidence in Mozambique and South Africa suggest the need for aggressive PrEP scale-up among all populations, due to an estimated HIV incidence higher than the mean, and PnR lower than the mean. In contrast, Eswatini, Lesotho, Namibia, Zambia, Botswana, Uganda, and Zimbabwe had estimated HIV incidence and PnR higher than the mean, suggesting the need for continued PrEP scale-up. All other countries reported an estimated HIV incidence below the average, but with higher PnR values; or they had both lower HIV incidence and PnR values than the average. This indicates the ongoing need to sustain PrEP programs to maintain the current progress toward achieving the UNAIDS 95-95-95 goals for HIV epidemic control (3).

Finally, the comparison of MER-derived and Naomi-derived PnR estimates resulted in higher Naomi-derived PnR estimates relative to MER-derived estimates, particularly among females (Supplementary Figures S2, S3). This was an unexpected finding, and we speculate that this may be due to PEPFAR's targeted approach for HIV testing that emphasizes case identification. The difference may be larger for females because of PEPFAR's targeted approach for priority populations includes the DREAMS (Determined, Resilient, Empowered, AIDS-free, Mentored and Safe) partnership to reduce the rate of HIV among AGYW in the highest HIV burden countries.

### Strengths and limitations

Strengths of this analysis include the use of routine, standardized programmatic data from a large number of countries, with the ability to disaggregate by age, sex, and key population. There are several limitations to this analysis inherent to PEPFAR MER results. First, MER data is reported cross-sectionally and in aggregate, thus individuals cannot be tracked longitudinally and there may be some duplication. Second, though all indicators are reported quarterly, only PrEP_NEW and HTS_TST_POS can be summed across quarters for a cumulative annual total. Because individuals can be reported under PrEP_CT in multiple quarters, this indicator cannot be summed for a cumulative total, which means that annual estimates of PnR may be underestimated. MER data are also subject to data quality and reporting completeness, including potential misclassification and measurement error. Though HTS_TST_POS was used a proxy for new diagnoses, this indicator measures the number of HIV-positive tests, rather than the number of individuals with a new HIV-positive diagnosis and includes an unquantified proportion of individuals previously diagnosed with HIV. To account for some of these limitations, Naomi-derived PnR estimates are included in the Supplementary Figure S1, however, estimated HIV incidence are for entire countries whereas MER PrEP data reflect only PEPFAR-supported geographies and sites within a country, which underestimates total PrEP use within a country. Additionally, PrEP_CT is a new MER indicator added in FY22 and may not be consistently reported across PEPFAR countries or sites. Furthermore, individuals re-initiating PrEP or transferring sites may be incorrectly reported as a new PrEP initiation (PrEP_NEW) rather than a continuing or re-initiating PrEP-user (PrEP_CT), which may overestimate PnR. Finally, key population status is self-reported, and social, structural, and legal barriers including criminalization, stigma, and discrimination likely under-report key populations and limit data availability.

Despite these limitations, this analysis provides a useful conceptual method to assess if PEPFAR-supported PrEP programming reflects population-level HIV prevention need and identify opportunities for scale-up. Although PEPFAR indicators have been instrumental for monitoring and evaluating PrEP introduction and scale-up, challenges persist in data collection, reporting, and interpretation due to complexities in defining and monitoring PrEP use relative to potential HIV exposure and risk (25–27). Unlike daily, life-long use of antiretroviral therapy required for people living with HIV, PrEP can be effective even when used episodically during periods of HIV risk for prevention-effective adherence (i.e., seasons of risk) (28–33). This methodology could be replicated using more granular or robust data from national or provincial government systems, particularly if examined alongside other epidemiological metrics such as ART coverage and population viral suppression (34).

PnR may be a useful metric for population PrEP coverage and unmet need, and can inform effective, data-driven, and person-centered prevention programs and policies. PrEP coverage can significantly reduce HIV incidence (8, 9), especially when offered alongside other biomedical HIV prevention options [e.g., condoms, post-exposure prophylaxis (PEP), and voluntary medical male circumcision (VMMC)] (35, 36). Person-centered, combination prevention that promotes dynamic prevention choice will have the greatest impact towards sustainable HIV control, as demonstrated by the SEARCH trial, in which dynamic choice between oral PrEP, injectable PrEP and PEP increased biomedical prevention coverage over five-fold and decreased HIV incidence to zero (37). Tailored strategies to scale and expand PrEP services by geography and subpopulation across the life course for those at greatest risk of HIV infection are crucial to achieving UNAIDS goal to end the AIDS epidemic as a public health threat for all by 2030 (38). It is essential to address inequities in PrEP access by eliminating regulatory barriers, fostering an enabling environment, promoting differentiated service delivery, and integrating PrEP with other health and social services to reduce stigma and normalize PrEP as a prevention option. At a community and individual level, increasing awareness and knowledge about PrEP is crucial, in which gain-framed messaging is aligned with individuals' perceptions of risk and prevention-effective adherence in which individuals cycle on and off or switch, as behaviors change, and as new prevention modalities become available (34).

## Conclusion

Progress towards ending the HIV/AIDS epidemic has stalled, with ongoing inequalities in access to HIV prevention that has resulted in an estimated 1.3 million people newly acquiring HIV in 2023. PEPFAR supports the majority of PrEP programming globally, and PnR can be a useful indicator of population PrEP coverage and unmet need, to inform effective, data-driven, and person-centered prevention programs and policies. This analysis underscores the need for PrEP scale-up and expansion to all geographies and key and priority populations for equitable access. This can be achieved by fostering enabling environments, promoting differentiated service delivery, and by supporting dynamic choice. Tailoring PrEP scale-up strategies by age, sex, key population, and geography is crucial to achieving UNAIDS targets and ending the AIDS epidemic as a public health threat for all by 2030.

## Data Availability

All data is publicly available at https://data.pepfar.gov/.
