# Supplementary material for: Disparities in PrEP use and unmet need across PEPFAR-supported programs: doubling down on prevention to put people first and end AIDS as a public health threat by 2030
Source: Front Reprod Health. 2024 Dec 13;6:1488970. doi: 10.3389/frph.2024.1488970 (PMC11671492; doi:10.3389/frph.2024.1488970)
Supplement: Supplementary file 1 [file Datasheet1.docx]

Table 1. MER-derived PrEP-to-need ratio (PnR) by country and population in PEPFAR-supported countries in FY23

|  |  |  | Female (age) | | | | Male (age) | | | | Key Populations | | | | |
| --- | --- | --- | --- | --- | --- | --- | --- | --- | --- | --- | --- | --- | --- | --- | --- |
| Region | Country | Overall N=48 | 15-24 N=48 | 25-34 N=48 | 35-49 N=48 | 50+ N=47 | 15-24 N=48 | 25-34 N=48 | 35-49 N=48 | 50+ N=48 | FSW N=45 | MSM N=46 | People in prisons N=13 | PWID N=26 | TG N=32 |
| Africa | Benin | 0.50 | 1.04 | 0.60 | 0.31 | 0.13 | 1.12 | 0.92 | 0.35 | 0.29 | 4.28 | 2.54 | -- | -- | -- |
|  | Botswana | 2.28 | 5.38 | 2.97 | 1.51 | 0.76 | 5.94 | 2.36 | 1.08 | 0.79 | 5.04 | 8.60 | 2.40 | 12.38 | 7.94 |
|  | Burkina Faso | 0.36 | 0.88 | 0.49 | 0.20 | 0.05 | 0.95 | 0.42 | 0.21 | 0.17 | 0.69 | 1.46 | -- | -- | -- |
|  | Burundi | 0.97 | 1.73 | 1.44 | 0.81 | 0.16 | 1.13 | 0.86 | 0.46 | 0.14 | 4.35 | 2.40 | -- | 1.26 | 9.25 |
|  | Cameroon | 0.18 | 0.25 | 0.23 | 0.14 | 0.04 | 0.69 | 0.30 | 0.05 | 0.01 | 3.17 | 1.80 | 0.01^§^ | * | 0.02^§^ |
|  | Cote d'Ivoire | 0.54 | 2.29 | 0.34 | 0.15 | 0.05 | 1.85 | 0.99 | 0.45 | 0.22 | 1.37 | 0.94 | † | 0.63^§^ | 0.29 |
|  | DRC | 0.56 | 0.98 | 0.66 | 0.38 | 0.15 | 1.78 | 0.97 | 0.42 | 0.25 | 2.41 | 1.69 | † | 14.88^§^ | 0.80 |
|  | Eswatini | 5.40 | 10.14 | 6.36 | 3.88 | 1.40 | 11.89 | 4.42 | 2.09 | 2.23 | 7.54 | 11.71 | 0.17 | 14.31 | 5.38 |
|  | Ethiopia | 0.75 | 3.23 | 1.12 | 0.19 | 0.03 | 0.15 | 0.15 | 0.07 | 0.07 | 4.08 | -- | 2.01 | -- | † |
|  | Ghana | 1.39 | 2.90 | 1.25 | 0.29 | 0.06 | 9.14 | 4.10 | 0.90 | 0.38 | 11.07 | 8.99 | † | -- | † |
|  | Kenya | 2.24 | 3.77 | 2.28 | 1.68 | 0.75 | 8.04 | 3.24 | 1.40 | 1.12 | 11.89 | 9.70 | 0.06 | 7.98 | 24.57 |
|  | Lesotho | 4.17 | 10.59 | 3.27 | 2.27 | 1.21 | 13.9 | 3.13 | 1.50 | 1.33 | 13.98 | 11.54 | 21.50 | 0.75^§^ | 1.64 |
|  | Liberia | 1.88 | 3.86 | 1.97 | 0.70 | 0.19 | 3.74 | 3.10 | 0.83 | 0.23 | 3.84 | 8.29 | -- | 54.13^§^ | 3.67 |
|  | Malawi | 1.10 | 2.68 | 0.98 | 0.81 | 0.50 | 4.05 | 1.36 | 0.61 | 0.56 | 5.26 | 8.64 | * | -- | 6.20 |
|  | Mali | 0.41 | 0.70 | 0.23 | 0.07 | * | 2.85 | 1.18 | 0.13 | 0.01 | 1.02 | 1.04 | -- | -- | -- |
|  | Mozambique | 0.57 | 1.38 | 0.51 | 0.37 | 0.13 | 1.85 | 0.47 | 0.3 | 0.23 | 3.00 | 3.82 | 1.95 | 2.33 | 3.60 |
|  | Namibia | 2.43 | 8.96 | 3.14 | 2.53 | 1.13 | 2.69 | 1.54 | 1.05 | 1.02 | 4.54 | 4.10 | -- | -- | 6.45 |
|  | Rwanda | 3.63 | 8.51 | 4.24 | 3.02 | 0.52 | 6.66 | 2.60 | 0.70 | 0.12 | 15.31 | 21.23 | 0.02 | * | † |
|  | Senegal | 0.27 | 0.53 | 0.31 | 0.17 | 0.07 | 0.86 | 0.53 | 0.10 | 0.03 | 1.91 | 0.88 | -- | -- | -- |
|  | Sierra Leone | 1.36 | 3.10 | 1.96 | 0.51 | 0.01 | 2.60 | 0.63 | 0.17 | 0.05 | 42.73^§^ | 60.63^§^ | -- | 47.12^§^ | -- |
|  | South Africa | 1.16 | 5.51 | 0.87 | 0.50 | 0.27 | 4.38 | 0.85 | 0.38 | 0.36 | 4.40 | 8.47 | 0.80 | 0.54 | 3.71 |
|  | South Sudan | 0.19 | 0.33 | 0.21 | 0.14 | 0.04 | 0.29 | 0.25 | 0.14 | 0.14 | 0.81 | -- | † | † | -- |
|  | Tanzania | 1.13 | 2.75 | 1.67 | 0.67 | 0.15 | 1.80 | 1.04 | 0.49 | 0.30 | 7.47 | 7.01 | 0.02 | 11.38 | † |
|  | Togo | 0.46 | 1.20 | 0.47 | 0.25 | 0.04 | 2.18 | 0.87 | 0.32 | 0.24 | 1.99 | 2.18 | -- | -- | -- |
|  | Uganda | 2.04 | 3.28 | 1.95 | 1.04 | 0.29 | 6.60 | 2.60 | 1.17 | 0.60 | 17.20 | 33.49 | 0.06 | 16.05 | 21.64 |
|  | Zambia | 2.35 | 4.38 | 1.78 | 1.32 | 0.75 | 8.95 | 3.11 | 1.50 | 1.24 | 6.97 | 12.17 | 13.83 | 8.50 | 11.76 |
|  | Zimbabwe | 1.92 | 3.82 | 1.67 | 1.60 | 1.01 | 5.25 | 1.73 | 0.92 | 0.92 | 6.17 | 7.72 | 2.00 | 9.81 | 2.82 |
| East Asia & Pacific | Burma | 0.57 | 0.14^§^ | 0.13^§^ | 0.07^§^ | 0.04^§^ | 1.26 | 0.48 | 0.23 | 0.16 | 0.13^§^ | 1.44 | -- | 0.01^§^ | 1.53 |
|  | Laos | 0.93 | 0.39^§^ | 0.26^§^ | 0.33^§^ | 0.20^§^ | 1.50 | 0.95 | 0.64 | 0.36 | * | 1.58 | -- | † | 0.99 |
|  | Philippines | 2.08 | 0.25^§^ | 0.14 | 0.35 | 0.50^§^ | 2.11 | 2.16 | 2.02 | 1.01 | 2.00^§^ | 2.61 | * | 0.06^§^ | 0.99 |
|  | Papua New Guinea | 0.11 | 0.04^§^ | 0.17 | 0.21 | 0.06^§^ | 0.13 | 0.10 | 0.08 | 0.18 | 0.24 | 0.39 | -- | -- | † |
|  | Thailand | 5.95 | 3.39 | 3.39 | 1.81 | 0.27 | 4.88 | 7.44 | 8.67 | 3.30 | 8.61 | 7.70 | † | 4.17 | 6.23 |
|  | Vietnam | 5.58 | 17.60 | 8.91 | 4.49 | 1.60 | 6.94 | 5.87 | 3.48 | 1.54 | 18.46 | 8.63 | * | 1.94 | 63.72 |

Table 1 (continued):

|  |  |  | Female | | | | Male | | | | Key Populations | | | | | |
| --- | --- | --- | --- | --- | --- | --- | --- | --- | --- | --- | --- | --- | --- | --- | --- | --- |
| Region | Country | Overall | 15-24 | 25-34 | 35-49 | 50+ | 15-24 | 25-34 | 35-49 | 50+ | FSW | MSM | People in  prisons | PWID | TG |  |
| Eastern  Europe & Central Asia | Kazakhstan | 0.89 | 1.45^§^ | 1.59 | 0.74 | 0.26 | 6.63 | 0.74 | 0.72 | 0.51 | † | 2.74 | -- | 0.89 | † |  |
|  | Kyrgyzstan | 1.71 | 1.88 | 1.35 | 0.81 | 0.53 | 4.71 | 1.69 | 1.43 | 1.44 | 2.25 | 5.14 | † | 8.06 | 14.50^§^ |  |
|  | Tajikistan | 1.61 | 5.25 | 2.71 | 1.95 | 0.85 | 3.38 | 0.93 | 0.99 | 1.20 | 10.43 | 23.65 | -- | 5.71 | * |  |
|  | Ukraine | 1.22 | 1.63 | 1.35 | 0.98 | 0.71 | 14.6 | 1.90 | 0.86 | 0.91 | 2.01 | 18.62 | † | 0.69 | † |  |
| Latin America & the Caribbean | Brazil | 6.46 | 3.19 | 3.07 | 1.24 | 0.34 | 4.00 | 7.82 | 9.98 | 10.40 | 1.72 | 8.62 | -- | 6.75 | 5.14 |  |
|  | Colombia | 0.07^§^ | 0.13^§^ | 0.07^§^ | 0.04^§^ | 0.03^§^ | 0.06^§^ | 0.08^§^ | 0.07^§^ | 0.02^§^ | 6.50^§^ | 0.23^§^ | -- | * | 0.50^§^ |  |
|  | Dominican Republic | 0.29 | 0.14 | 0.12 | 0.12 | 0.04 | 1.21 | 0.66 | 0.27 | 0.09 | 0.75 | 4.02 | -- | 0.91^§^ | 1.86 |  |
|  | El Salvador | 3.49 | 0.14^§^ | 0.19 | 0.04 | 0.01^§^ | 5.00 | 5.15 | 2.87 | 0.73 | 0.42 | 9.57 | * | -- | 27.88 |  |
|  | Guatemala | 1.93 | 2.24 | 1.52 | 0.82 | 0.11 | 2.15 | 2.61 | 1.81 | 0.36 | 38.92 | 4.28 | * | * | 6.52 |  |
|  | Haiti | 1.06 | 1.21 | 0.76 | 0.36 | 0.10 | 7.60 | 2.54 | 0.69 | 0.19 | 4.50 | 7.78 | * | 6.05 | 16.06^§^ |  |
|  | Honduras | 2.31 | 1.85 | 1.86 | 1.13 | 0.41 | 4.52 | 2.76 | 1.82 | 0.53 | 152.88^§^ | 6.40 | -- | -- | 8.62 |  |
|  | Jamaica | 1.48 | 0.58 | 1.39 | 0.34 | 0.25 | 1.73 | 2.28 | 1.11 | 1.60 | -- | 0.86 | † | -- | * |  |
|  | Panama | 1.07 | 0.20 | 0.22 | 0.25 | 0.09 | 0.96 | 1.64 | 1.33 | 0.68 | 8.17 | 4.07 | -- | -- | 1.38 |  |
| South Asia | India | 0.12 | 0.61 | 0.33 | 0.06 | 0.01 | 0.16 | 0.16 | 0.04 | 0.01 | 0.50 | 0.97 | -- | <0.01^§^ | 8.08 |  |
|  | Nepal | 4.83 | 14.03 | 9.47 | 4.77 | 0.27 | 8.24 | 3.94 | 2.30 | 0.70 | 22.96 | 7.18 | -- | -- | 9.02 |  |
| -- Unable to calculate PnR due to no PrEP and HTS data  * Unable to calculate PnR due to no PrEP data  † Unable to calculate PnR due to no HTS data  ^§^ Interpret PnR estimate with caution due to incomplete data (no PrEP and/or HTS results for 3 quarters)  *PnR estimates not calculated for Nigeria due to ongoing data quality improvement efforts in FY23. MER results for PrEP were not reported for Cambodia or Indonesia and in FY23*  Abbreviations: FSW (female sex workers), MSM (men who have sex with men), PWID (people who inject drugs), TG (transgender) | | | | | | | | | | | | | | | |  |

Table 2. Naomi-derived PrEP-to-need ratio (PnR) by country and age/sex in PEPFAR-supported countries in FY23

|  |  | Female (age) | | | | Male (age) | | | |
| --- | --- | --- | --- | --- | --- | --- | --- | --- | --- |
|  | Overall | 15-24 | 25-34 | 35-49 | 50+ | 15-24 | 25-34 | 35-49 | 50+ |
| Benin | 1.97 | 1.32 | 2.35 | 2.32 | 0.88 | 2.16 | 2.08 | 2.65 | 4.37 |
| Botswana | 4.68 | 4.89 | 5.38 | 5.31 | 2.22 | 4.15 | 4.02 | 4.33 | 3.66 |
| Burkina Faso | 1.66 | 2.00 | 2.04 | 1.39 | 0.43 | 1.59 | 1.12 | 1.43 | 2.40 |
| Burundi | 5.88 | 6.67 | 10.27 | 7.43 | 2.62 | 1.80 | 2.85 | 3.39 | 2.74 |
| Cameroon | 1.34 | 0.64 | 1.80 | 1.56 | 0.44 | 2.33 | 2.21 | 0.94 | 0.24 |
| Cote d'Ivoire | 1.86 | 3.11 | 1.10 | 0.75 | 0.30 | 7.43 | 2.05 | 1.58 | 1.12 |
| DRC | 3.48 | 2.06 | 3.32 | 3.66 | 1.31 | 4.16 | 4.67 | 5.97 | 4.02 |
| Eswatini | 10.57 | 10.79 | 12.71 | 12.10 | 2.57 | 10.32 | 8.57 | 9.49 | 11.94 |
| Ethiopia | 4.45 | 8.28 | 7.08 | 1.94 | 0.22 | 0.40 | 0.72 | 0.74 | 0.93 |
| Ghana | 0.61 | 0.55 | 0.56 | 0.19 | 0.06 | 2.10 | 1.09 | 0.48 | 0.31 |
| Haiti | 0.34 | 0.63 | 0.41 | 0.61 | 2.11 | 0.09 | 0.18 | 0.51 | 2.05 |
| Kenya | 12.06 | 8.88 | 13.45 | 13.73 | 6.25 | 12.68 | 14.13 | 16.28 | 20.74 |
| Lesotho | 12.12 | 18.54 | 10.65 | 11.38 | 4.43 | 11.82 | 7.26 | 8.06 | 8.19 |
| Liberia | 10.90 | 6.84 | 13.50 | 6.34 | 1.28 | 16.98 | 20.90 | 10.38 | 3.31 |
| Malawi | 6.48 | 9.15 | 4.42 | 5.32 | 4.40 | 19.14 | 5.59 | 3.64 | 3.94 |
| Mali | 0.75 | 0.42 | 0.54 | 0.27 | * | 1.93 | 1.62 | 0.33 | 0.03 |
| Mozambique | 2.21 | 2.56 | 2.72 | 1.31 | 0.30 | 2.87 | 2.41 | 1.67 | 1.02 |
| Namibia | 6.27 | 8.43 | 5.95 | 9.88 | 4.10 | 1.93 | 2.84 | 5.21 | 5.79 |
| Rwanda | 8.68 | 13.35 | 11.97 | 8.59 | 1.17 | 7.08 | 5.49 | 2.24 | 0.48 |
| Senegal | 0.54 | 1.51 | 0.96 | 1.17 | 2.11 | 1.40 | 0.38 | 0.08 | 0.13 |
| Sierra Leone | 3.74 | 4.66 | 9.53 | 2.26 | 0.04 | 2.02 | 1.94 | 0.65 | 0.13 |
| South Africa | 3.13 | 6.31 | 2.74 | 1.30 | 0.36 | 4.10 | 1.99 | 1.51 | 0.94 |
| Tanzania | 8.03 | 8.84 | 13.28 | 6.81 | 1.56 | 3.16 | 7.04 | 6.05 | 3.30 |
| Togo | 1.77 | 1.09 | 1.53 | 1.84 | 0.51 | 4.87 | 1.95 | 1.79 | 2.35 |
| Uganda | 6.82 | 6.43 | 7.65 | 4.63 | 1.20 | 9.46 | 7.76 | 6.80 | 4.63 |
| Zambia | 11.38 | 10.00 | 9.98 | 11.05 | 6.04 | 14.14 | 13.68 | 14.27 | 13.71 |
| Zimbabwe | 9.16 | 11.70 | 8.10 | 11.18 | 7.94 | 8.54 | 6.03 | 7.33 | 10.37 |
| * Unable to calculate PnR due to incomplete PrEP data  Naomi estimates only available by age/sex for countries shown. PnR estimates were not calculated for Nigeria due to ongoing data quality improvement efforts in FY23. | | | | | | | | | |

Figure 1. Relationship between HIV incidence and Naomi-derived PrEP-to-need ratio (PnR) in select PEPFAR-supported countries in FY23


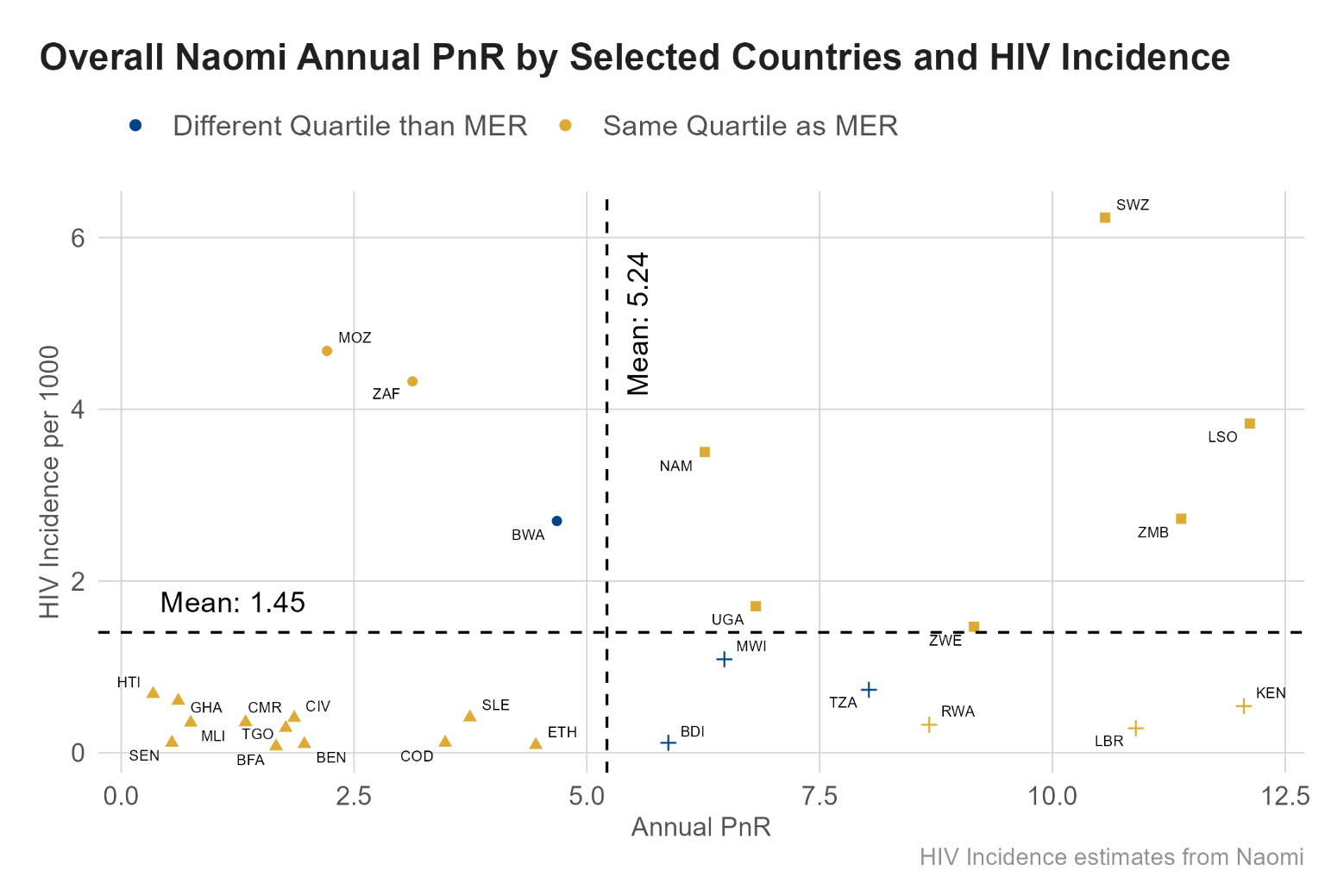


*Note: Different quartile when compared to MER-derived PnR show in Figure 4.* *Data is limited to countries with available HIV incidence from Naomi (*UNAIDS epidemiological estimates, 2024 (<https://naomi-spectrum.unaids.org>))

Abbreviations: Benin (BEN), Botswana (BWA), Burkina Faso (BFA), Burundi (BDI), Cameroon (CMR), Cote d’Ivoire (CIV), Democratic Republic of Congo (COD), Eswatini (SWZ), Ethiopia (ETH), Ghana (GHA), Haiti (HTI), Kenya (KEN), Lesotho (LSO), Liberia (LBR), Malawi (MWI), Mali (MLI), Mozambique (MOZ), Namibia (NAM), Rwanda (RWA), Senegal (SEN), Sierra Leone (SLE), South Africa (ZAF), Togo (TGO), United Republic of Tanzania (TZA), Uganda (UGA), Zambia (ZMB), and Zimbabwe (ZWE)

Figure 2. Distribution of MER-derived and Naomi-derived PrEP-to-need ratios (PnR) by age among females in FY23


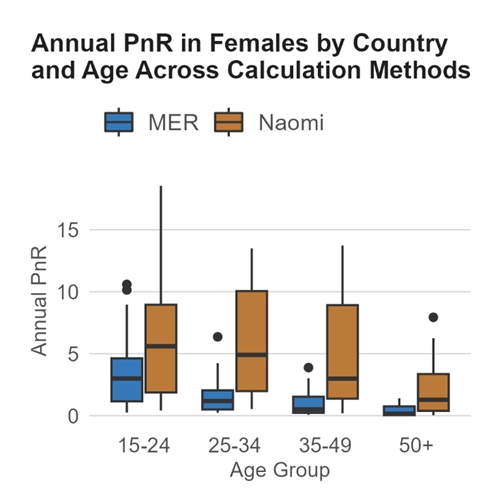


*Note: A comparison of PnR estimates derived from MER and from Naomi are only available for 27 countries (Benin, Botswana, Burkina Faso, Burundi, Cameroon, Cote d'Ivoire, DRC, Eswatini, Ethiopia, Ghana, Haiti, Kenya, Lesotho, Liberia, Malawi, Mali, Mozambique, Namibia, Rwanda, Senegal, Sierra Leone, South Africa, Togo, Uganda, United Republic of Tanzania, Zambia, Zimbabwe)*

Figure 3. Distribution of MER-derived and Naomi-derived PrEP-to-need ratios (PnR) by age among males in FY23


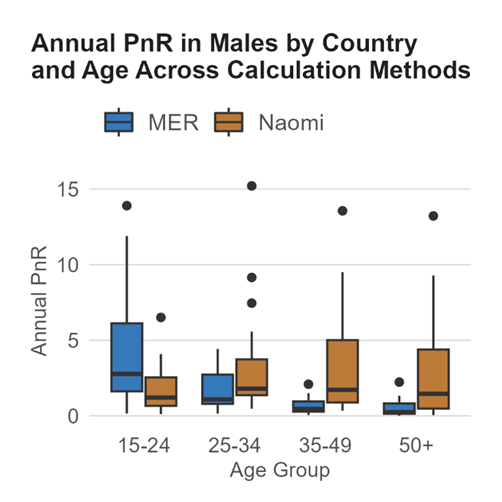


*Note: A comparison of PnR estimates derived from MER and from Naomi are only available for 27 countries (Benin, Botswana, Burkina Faso, Burundi, Cameroon, Cote d'Ivoire, DRC, Eswatini, Ethiopia, Ghana, Haiti, Kenya, Lesotho, Liberia, Malawi, Mali, Mozambique, Namibia, Rwanda, Senegal, Sierra Leone, South Africa, Togo, Uganda, United Republic of Tanzania, Zambia, Zimbabwe)*
